# Evaluation of ocular pulse amplitude in non-arteritic anterior ischaemic optic neuropathy

**DOI:** 10.1186/s12886-017-0430-6

**Published:** 2017-03-29

**Authors:** Durgul Acan, Omer Karti, Tuncay Kusbeci

**Affiliations:** 1 Bozyaka Training and Research Hospital, Department of Ophthalmology, Izmir, Turkey; 2 Ilica Mah Prof Dr Turkan Saylan Cad No:41/15, 35320, Narlidere, Izmir Turkey

**Keywords:** Dynamic contour tonometry, Intraocular pressure, Ocular blood flow, Optic nerve, Short posterior ciliary arteries

## Abstract

**Background:**

To evaluate the ocular pulse amplitude (OPA) in patients with chronic non-arteritic anterior ischaemic optic neuropathy (NAION).

**Methods:**

This cross-sectional study comprised a study group of 30 eyes from 30 patients with NAION and a control group of 31 eyes from 31 age and gender-matched healthy subjects. Bilateral OPA was measured with dynamic contour tonometry (DCT) and was compared between the study and control groups.

**Results:**

No statistically significant difference was found between the study and control groups in terms of hypertension, diabetes mellitus, ischaemic heart disease and hyperlipidemia. The mean intraocular pressure (IOP) measured with Goldmann Applanation Tonometry and DCT in the study and control groups was not statistically different (p_1_ = 0.094, p_2_ = 0.240). The mean OPA in the study group and the control group were 2.01 ± 0.69 mmHg and 1.97 ± 0.68 mmHg (*p* = 0.839).

**Conclusion:**

No significant difference was determined in the OPA levels of eyes with NAION at the chronic stage and eyes in the control group.

## Background

Anterior ischaemic optic neuropathy (AION) is a consequence of ischaemic injury to the anterior section of the optic nerve, which is an area of circulation fed by the posterior ciliary arteries. There are 2 types of AION: arteritic AION (AAION), which develops secondary to vasculitis (primarily giant cell arteritis), and non-arteritic AION (NAION), which develops secondary to non-inflammatory small vessel disease [1–3]. NAION constitutes 95% of all AION and in those aged >50 years, is the most frequent cause of acute optic neuropathy. The annual incidence among those aged >68 years has been estimated to be 82 per 100,000 and 0.54 per 100,000 for all age groups [4, 5].

It is thought that NAION results from inadequate circulation within the optic nerve head, although there has not yet been any conclusive proof of the specific location of the vasculopathy and the pathogenetic mechanism [6]. Normally, blood flow remains stable by perfusion pressure, intraocular pressure (IOP) and metabolic conditions (including tissue oxygen and carbondioxide levels). Arteriosclerosis, vasospasm, or medications such as beta-blockers or other antihypertensive drugs can disrupt the autoregulatory mechanism. In patients with NAION, there is reduced blood flow in the short posterior ciliary arteries (SPCAs) [7], although there has been no histopathological evidence of thrombosis of the SPCAs in the very few cases that have been studied [8, 9]. Therefore, it is believed that the condition is caused by generalized hypoperfusion in some cases and by occlusion of disc or laminar capillaries in others.

Extended pulsatile oscillations of IOP during the cardiac cycle are represented by ocular pulse amplitude (OPA). In cases of giant cell arteritis, OPA has been demonstrated to be significantly reduced, and may therefore be helpful in the prediction of NAION risk [10–13].

The aim of the present study was to evaluate OPA in patients with NAION at the chronic stage.

## Methods

A review was made of patients diagnosed with NAION, who were admitted to the Ophthalmology Clinic at Bozyaka Training and Research Hospital, Izmir, Turkey between 2011 and 2015. The study group included a total of 30 eyes of 30 patients who attended the clinic for a routine check-up between June 2014 and October 2015 and a control group included a total of 31 patients who attended the clinic for a refraction examination. The medical records of all patients were reviewed and a comparison was made of the study group and the control group. The study was conducted in accordance with the tenets of the Declaration of Helsinki, with approval from the Local Ethics Committee of this hospital. Informed consent for participation in this study was obtained from each participant.

The criteria required for diagnosis of NAION were as follows: (1) a history of sudden visual loss and an absence of other ocular, orbital, systemic, or neurological diseases that may influence or explain the patient’s visual symptoms; (2) presence of optic disc edema at the time the patient came to medical attention shortly after the onset of acute symptoms; (3) spontaneous resolution of optic disc edema was observed; and (4) the eye had optic disc-related visual field defects.

Patients were excluded if they had media opacities that would preclude fundus examination or visual field evaluation, glaucoma, coexistence of ophthalmic or neurological disease, or any other retinal pathology, or if they had undergone previous eye surgery other than uneventful cataract surgery. Patients with diabetes mellitus were included, but those who had vitreous hemorrhages, traction detachment, or other complications influencing visual acuity or visual fields were excluded. Eyes with unreliable visual fields were excluded. In addition, patients who were in the acute stage of NAION were excluded from the study, in order to minimise the effects of optic disc swelling.

The control group consisted of subjects with no history of chronic ocular or systemic disease or of ocular surgery other than uneventful cataract surgery. The inclusion criteria for this group were that they had a normal visual field test, IOP of less than 21 mmHg, a normal, symmetric optic disc head between left and right eyes, an open anterior chamber angle, no history of chronic, ocular or systemic corticosteroid use, spherical refraction of < ±5.00 dioptres and astigmatism of < ±3.00 dioptres.

A detailed medical history was taken from each participant including all previous or current systemic diseases, with particular focus on arterial hypertension, diabetes mellitus, ischaemic heart disease and hyperlipidemia. Blood pressure measurements were taken using a brachial sphygmomanometer (DS66, Welch Allyn, Skaneateles, NY, USA) and a Littmann Classic II stethoscope (3 M, St Paul, MN, USA) on the upper right-side arm after a rest period of at least 5 minutes. Patients were told to abstain from caffeine, exercise, and smoking for at least 30 minutes before the examination. The measurements were repeated after a 5-minute interval and the average of the two values was recorded. The height (in centimeters) and weight (kilograms) of the patients were measured using a standard scale, and in light clothing and bare feet, and noted. Body Mass Index (BMI) was calculated using the formula weight (kg)/height squared (m^2^).

A comprehensive ophthalmic evaluation was performed at that time, which included (1) recording of the best-corrected visual acuity (BCVA) using a Snellen chart, (2) the visual field measurement using a Humphrey Automated Field Analyzer (Carl Zeiss Meditec Inc, Dublin, CA, USA) program 30–2 and the standard Swedish interactive threshold algorithm (SITA) strategy, (3) slit-lamp examination of the anterior segment, lens, and vitreous, (4) IOP with Goldmann applanation tonometry (GAT) and dynamic contour tonometry (DCT, PASCAL, Swiss Microtechnology AG, a Ziemer Ophthalmic Systems Group Company), (5) OPA with DCT, (6) The central corneal thickness (CCT) was measured three times using ultrasonic pachymetry with a Pacscan 300P USP device (Sonomed Inc., Lake Success, NY, USA) (6), direct and indirect ophthalmoscopy and (7) color fundus photography. OPA measurements were taken using a slit lamp-mounted DCT device at the same time of day (09:00–10:00) in all cases, thus avoiding diurnal fluctuations. All the measurement procedures were applied by the same two experienced ophthalmologists (DA and OK) according to the manufacturer’s recommendations. Only Quality index (‘Q’) readings of 1 or 2 (range: 1–5, with higher values indicating lower quality) were included in the analysis. The ‘OPA’ value of each eye was calculated as the mean of three consecutive readings. Both eyes were examined in the study and control groups but only the values of the right eyes of the control group were used for analyses.

Statistics were analyzed using SPSS software version 15.0 (SPSS, Inc, Chicago, Illinois, USA). The data were given as mean values ± SDs. The Gaussian distribution of the parameters was tested using the Kolmogorov–Smirnov test. Continuous variables were compared between the groups using the Student t-test and categorical variables were compared using the χ2 test. The Pearson Chi-square test with Yates continuity correction or the Fisher exact test, as appropriate, were used for frequency distribution comparisons. All tests were two tailed, and significance was set at 0.05.

## Results

The study group included 30 eyes of 30 patients (14 males, 16 females) with NAION. The mean age was 54.67 ± 10.54 years, the mean refractive error was +0.38 ± 1.43 (range, −3.75 to +4.00) diopters and the mean follow-up was 2.01 years (range, 0.30 – 10 years) in the study group. Two patients had bilateral NAION and their right eyes were evaluated. The control group included 31 eyes of 31 patients (10 males, 21 females) with no abnormality of the fundus. The mean age in the control group was 53.39 ± 6.67 years, and the mean refractive error was +0.47 ± 1.12 (range, −3.50 to +3.25) diopters. No statisically significant difference was determined between the study group and the control group in respect of refractive error, age and gender (p_1_ = 0.788, p_2_ = 0.575 and p_3_ = 0.249, respectively). Descriptive statistics for the demographic, ocular and systemic characteristics of the study and control groups are shown in Table 1. Compared to the control group, patients with NAION had a greater prevalence of hypertension (40.0 vs. 29.0%; *p* = 0.367), diabetes mellitus (30.0 vs. 22.6%; *p* = 0.510), ischaemic heart disease (13.3 vs 9.7%; *p* = 0.707) and hyperlipidemia (26.7 vs. 12.9%; *p* = 0.176) but the difference was not statistically significant. The mean BMI values in the study and control groups were 29.08 ± 5.23 and 27.77 ± 4.67, respectively (*p* = 0.304). The mean systolic blood pressure (SBP) in the study group and the control group was 122.83 ± 13.11 mmHg and 118.71 ± 15.97 mmHg, respectively (*p* = 0.276). The mean diastolic blood pressure (DBP) was also not statistically different (77.70 ± 7.66 mmHg in the study group vs. 75.00 ± 11.25 mmHg in the control group; *p* = 0.279).

**Table 1:** Demographic, ocular and systemic characteristics of the tested subjects

	Study group	Control group	*p* value
	(*n* = 30 eyes)	(*n* = 31 eyes)	
Mean age (year ± SD)	54.67 ± 10.54	53.39 ± 6.67	0.575
Gender, no. of females (%)	16 (53.3%)	21 (67.7%)	0.249
BMI, mean (SD), kg/m^2^	29.08 ± 5.23	27.77 ± 4.67	0.304
Hypertension n,(%)	12 (40.0%)	9 (29.0%)	0.367
SBP, mmHg	122,83 ± 13.11	118.71 ± 15.97	0.276
DBP, mmHg	77.70 ± 7.66	75.00 ± 11.25	0.279
Ischaemic heart disease n, (%)	4 (13.3%)	3 (9.7%)	0.707
DM n, (%)	9 (30.0%)	7 (22.6%)	0.510
Hyperlipidemia n, (%)	8 (26.7%)	4 (12.9%)	0.176
BCVA	0.35 ± 0.37	0.96 ± 0.09	<0.001
Spheric equivalant, mean	0.38 ± 1.43	0.47 ± 1.12	0.788
Mean GAT, mmHg ± SD	13.73 ± 2.30	14.84 ± 2.74	0.094
Mean DCT, mmHg ± SD	13.58 ± 1.81	14.20 ± 2.22	0.240
Mean OPA, mmHg ± SD	2.01 ± 0.69	1.97 ± 0.68	0.839
CCT, μm	536.47 ± 35.04	546.61 ± 30.03	0.229

*Abbreviations BCVA* best-corrected visual acuity, *BMI* body-mass index, *CCT* central corneal thickness, *DBP* diastolic blood pressure, *DCT*, dynamic contour tonometer, *DM* diabetes mellitus, *GAT* Goldmann applanation tonometry, *OPA* ocular pulse amplitude, *SBP* systolic blood pressure.

**Fig. 1:**
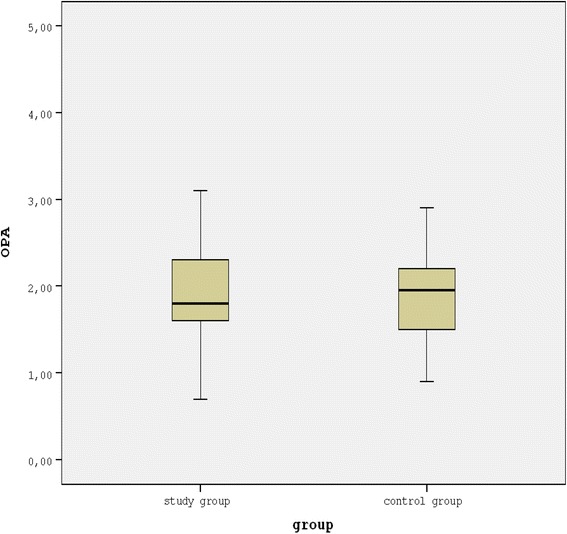
Boxplot showing the mean OPA in the study group and the control group

The mean BCVA measured with a Snellen chart was 0.35 ± 0.37 in the study group and 0.96 ± 0.09 in the control group (*p* < 0.001). In the study group, the time since the NAION attack ranged from 4 months to 10 years (mean, 2.01 years). The mean IOP with GAT in the study and control groups was 13.73 ± 2.30 mmHg and 14.84 ± 2.74 mmHg, respectively (*p* = 0.094). The IOP with DCT was 13.58 ± 1.81 mmHg and 14.20 ± 2.22 mmHg, respectively for the study and control groups (*p* = 0.240). The mean OPA in the study group and the control group was 2.01 ± 0.69 mmHg and 1.97 ± 0.68 mmHg (*p* = 0.839) (Fig. 1). The OPA measurements were compared between the affected eyes and the contralateral unaffected eyes within the group of patients with unilateral NAION and without histories of previous NAION in the contralateral eye. The mean OPA values in the affected eyes of the unilateral NAION (28 eyes) were significantly lower than the unaffected contralateral eyes (1.95 ± 0.67 mmHg and 2.15 ± 0.61 mmHg, respectively, *p* = 0.035, Wilcoxon Signed Ranks Test). However, the difference between the mean OPA of the affected eyes of the unilateral NAION and the mean OPA of the control group was not statistically significant (*p* = 0.857).

## Discussion

In this study, the OPA values in patients with NAION were investigated and it was also examined whether OPA was an indicator for identifying eyes with NAION from healthy eyes. OPA with DCT is a non-invasive, safe, fast and readily available procedure, therefore, in most cases, it can be easily integrated into the diagnostic work-up [14, 15]. Typically, reduced OPA values are measured in carotid artery stenosis or encircling buckles for retinal detachment [16, 17]. OPA is thought to be a potential alternative parameter for ocular blood flow measurement and although there have been studies of glaucoma association [18], there has not been much research related to NAION. In a study by Weizer et al., OPA was found to be correlated with the severity of glaucoma, with high OPA seeming to indicate less severe glaucoma [19]. Schwenn et al. demonstrated that eyes with normal tension glaucoma were more likely to have low OPA than those with primary open angle glaucoma, ocular hypertension, or normal eyes [20]. Kynigopoulos et al. reported that low OPA was related to functional and structural damage in primary open angle glaucoma [21]. In contrast, the results of a study by Figueiredo et al., showed significantly lower mean OPA in the control group than in the patients with glaucoma or ocular hypertension [22].

Another study compared OPA in patients with giant cell arteritis and those with NAION or non-arteritic central retinal artery occlusion [13] and the mean pulse amplitude of patients with AAION was determined to be only 37% of the mean value of the non-arteritic group. In a different study, the mean OPA with pneumotonometer was seen to be reduced in patients with temporal arteritis but not in cases where AION was not caused by temporal arteritis [12]. However, these studies are old and there is no current study with new techniques, such as DCT, for comparision of NAION and healthy eyes.

Knecht et al. evaluated the OPA values of the eyes of 31 patients with suspected giant cell arteritis and confirmed the association of reduced OPA levels in giant cell arteritis. The findings were also supportive of an algorithm based on OPA, erythrocyte sedimentation rate and thrombocyte count, which has the potential to provide valuable assistance in the clinical management of giant cell arteririts [23]. As OPA has been shown to be markedly reduced in giant cell arteririts, the hypothesis of the current study was that it may be helpful in predicting the likelihood of NAION [10–13].

In this study, OPA was compared between patients with NAION and a healthy control group. There was no difference between the two groups in terms of gender, age, spherical equivalent refraction and systemic characteristics. The OPA values in NAION were not found to be any different from those of the eyes in the control group. However, the mean OPA values in the affected eyes of the patients with unilateral NAION were statistically lower than those of the unaffected contralateral eyes. The increase in OPA values of the unaffected contralateral eyes might have developed psychologically to protect the unaffected eye, but the mechanism is unclear. In a previous study, eyes affected by NAION and unaffected contralateral eyes showed significantly thinner macular choroids than eyes of a control group after adjusting ocular and systemic parameters [24]. It was claimed that a thin macular choroid may thus potentially be a risk factor for the development of NAION and it may be an indicator for changed blood circulation at the posterior pole [24]. Blood supply to both the choroid and the anterior section of the optic nerve head is provided by the SPCAs [25]. Therefore, a chronic, but balanced, vascular insufficiency in these arteries could be considered to decompensate with an acute event in the optic nerve head region such as an overnight capillary occlusion together with a nocturnal decrease in arterial blood pressure [24]. However, Hayreh reported that the choroidal vascular bed (except for the small peripapillary choroid), has no role in the blood supply of the optic nerve head [26]. The total blood flow in the choroid and changes in it do not give a true picture of the blood flow in the optic nerve head. Total ocular blood flow has pulsatile and nonpulsatile components. In a study by Arnold et al. choroidal fluorescein flow was normal in eyes with NAION, indicating no restriction in the choroidal blood supply [27]. However, in experimental studies, optic nerve capillaries have been shown to be largely derived from feeder branches derived from the choroidal vasculature surrounding the optic nerve [28]. Hayreh stated that NAION is a hypotensive disorder, arising from transient noctural non-perfusion or hypoperfusion of the blood vessels in the optic nerve head, and is not caused by occlusion of the SPCAs as has been frequently previously claimed [29]. The lack of consistent choroidal filling delay demonstrated in fluorescein angiography studies of NAION has suggested that the impaired perfusion originates in the paraoptic tributaries of the SPCAs, which are distal to the split from the choroidal branches [30]. This, therefore, could explain the lack of any change in the OPA results in the current study.

There are some limitations of the current study. Pourjavan et al. found that in normal healthy eyes, the OPA remained stable during normal outpatient office hours and was not correlated with the blood pressure of patients [31]. However, in eyes with NAION or at risk of NAION, this strategy may not be valid. Nocturnal hypotension could have an effect on OPA values. The other limitation was the low number of patients. Although efforts were made to recruit as many samples as possible, it is accepted that the small sample size of this study may cause a weakness in the statistical results.

## Conclusions

OPA levels in the eyes with NAION were not found to be significantly different from those of the control eyes. This result could be clinically important in the differentiation between NAION and AAION at the chronic stage, in accordance with previous studies that have shown reduced OPA levels in AAION. However, prospective studies with larger sample sizes are needed to confirm these findings.

## Abbreviations

AAION﻿: Arteritic anterior ischaemic optic neuropathy
AION: Anterior ischaemic optic neuropathy
BCVA: Best-corrected visual acuity
BMI: Body mass index
CCT: Central corneal thickness
DBP: Diastolic blood pressure
DCT: Dynamic contour tonometry
GAT: Goldmann applanation tonometry
IOP: Intraocular pressure
NAION: Non-arteritic ischaemic optic neuropathy
OPA: Ocular pulse amplitude
SBP: Systolic blood pressure
SITA: Swedish interactive threshold algorithm
SPCAs: Short posterior ciliary arteries
